# MRI evaluation of axillary and intramammary lymph nodes in the postoperative period

**DOI:** 10.1111/tbj.13355

**Published:** 2019-06-07

**Authors:** Joao V. Horvat, Elizabeth A. Morris, Blanca Bernard‐Davila, Danny F. Martinez, Doris Leithner, Rosa Elena Ochoa‐Albiztegui, Sunitha B. Thakur, Katja Pinker

**Affiliations:** ^1^ Department of Radiology Memorial Sloan Kettering Cancer Center New York New York; ^2^ Department of Diagnostic and Interventional Radiology University Hospital Frankfurt Frankfurt am Main Germany; ^3^ Department of Medical Physics Memorial Sloan Kettering Cancer Center New York New York; ^4^ Division of Molecular and Gender Imaging, Department of Biomedical Imaging and Image‐guided Therapy Medical University of Vienna Wien Austria; ^5^ Present address: Department of Radiology University Hospital, University of São Paulo São Paulo Brazil

**Keywords:** breast neoplasms, lymph nodes, magnetic resonance imaging, postoperative period, recurrence

## Abstract

Our study aimed to evaluate if breast‐conserving surgery and adjuvant treatment could affect the morphological features of axillary and intramammary lymph nodes on magnetic resonance imaging (MRI) in patients with invasive breast cancer and clinically negative axilla. In this single‐center study, we retrospectively evaluated 50 patients who had (a) breast‐conserving surgery, (b) clinically negative axilla, (c) preoperative MRI within 3 months before surgery, and (d) postoperative MRI within 12 months after surgery. Axillary and intramammary lymph nodes on postoperative MRI were identified and then compared with preoperative MRI by two breast radiologists with regards to the following: enlargement, cortical thickening, presence of fatty hilum, irregularity, heterogeneity, matting, and axillary lymph node asymmetry. Three hundred and two axillary and eight intramammary lymph nodes were evaluated. Enlargement and cortical thickening were seen in 5/50 (10%) patients in three axillary and two intramammary lymph nodes. None of the lymph nodes on postoperative MRI demonstrated occurrence of lack of fatty hilum, irregularity, heterogeneity, matting or axillary lymph node asymmetry. No evidence of recurrence was observed on 2‐year follow‐up. Lymph node enlargement and cortical thickening may be observed in a few patients in the postoperative period. Nevertheless, in patients with clinically negative axilla, these changes in morphology are often related to treatment rather than malignancy and favor short‐term follow‐up as an alternative to lymph node biopsy.

## 1 INTRODUCTION

Nodal status in breast cancer is one of the determining factors for staging, treatment, and prognosis. Axillary lymph node (LN) dissection is the traditional surgical approach for assessment of nodal staging. In the last two decades, sentinel LN biopsy has become the method of choice for selecting patients with negative LNs in whom axillary LN dissection can be avoided, reducing the incidence of postsurgical complications.^1^ More recently, the American College of Surgeons Oncology Group Z0011 study demonstrated that axillary LN dissection can also be avoided in patients with 1‐2 positive LNs on sentinel LN biopsy if they meet certain criteria.^2^, ^3^ Although there are studies that demonstrate that preoperative imaging of LNs is still necessary, some authors believe that the importance of preoperative evaluation of LNs has been diminished.^4^, ^5^, ^6^, ^7^

While these advances impact nodal staging in the preoperative setting, there is a need to also advance the assessment of LNs following breast‐conserving surgery to determine the possibility of recurrence. In this setting, patients treated for breast cancer will be followed with imaging. While some patients will be followed with mammography and ultrasound only, others will also undergo magnetic resonance imaging (MRI) to rule out recurrence.^8^, ^9^, ^10^ In comparison with ultrasound and mammography, MRI not only has a higher sensitivity for detecting recurrence in the breast but is also able to visualize some LNs that are not assessible on other modalities.^11^

Several studies have investigated MRI for LN assessment in breast cancer.^12^, ^13^, ^14^, ^15^, ^16^, ^17^, ^18^, ^19^, ^20^, ^21^, ^22^, ^23^, ^24^ Whereas morphology may be preserved in some LNs with metastatic infiltration, benign processes like inflammatory response may also cause significant changes in LN morphology. To this date, there is no consensus in the literature about which MRI parameters should be used to raise suspicion. Nonetheless, the imaging features such as presence of enlargement, cortical thickening, lack of fatty hilum, irregular contours, matting, and axillary nodal asymmetry have been reported to be associated with malignant infiltration.^15^, ^16^, ^17^, ^18^, ^19^, ^20^, ^21^, ^22^

Moreover, the investigation of LNs in breast cancer has mainly focused on the pretreatment setting^12^, ^13^, ^14^, ^15^, ^16^, ^17^, ^18^, ^19^, ^20^, ^21^, ^22^, ^23^, ^24^ and their imaging features on MRI in the postoperative period have not been fully explored. Changes in imaging features may be benign sequelae of surgery and radiation therapy, or may be related to postsurgical complications, such as infection that can be difficult to distinguish from recurrent or metastatic disease.^25^

In this context, the aim of this study was to evaluate if breast‐conserving surgery and adjuvant treatment affect the morphological features of axillary and intramammary LNs on MRI in patients with invasive breast cancers and negative axillae.

## 2 MATERIALS AND METHODS

The Institutional Review Board approved this single‐center Health Insurance Portability and Accountability Act compliant retrospective study and waived the requirement for patient informed consent.

### 2.1 Patients

The institutional data base was queried for consecutive patients from January 2010 to December 2015 who matched the following criteria: (a) breast‐conserving surgery for invasive breast cancer, (b) clinically negative axilla with negative sentinel LN biopsy, (c) preoperative MRI within 3 months before surgery, and (d) postoperative MRI within 12 months after surgery. Exclusion criteria were (a) poor imaging quality, (b) axillary LN dissection, and (c) neoadjuvant treatment for breast cancer. Fifty patients were included in the study with one patient presenting with bilateral breast cancer.

### 2.2 Data analysis

The information obtained from medical records was reviewed for patient age, date and type of surgery, dates of preoperative and postoperative MRI studies, and adjuvant treatments received including radiation, chemo and hormone therapies. Preoperative and follow‐up consultations and imaging reports were also reviewed for evidence of nodal metastatic disease or recurrence.

### 2.3 Histopathology

The histopathology findings from the surgical specimens of the primary tumor were considered as the standard of reference. Reports were reviewed for tumor type and immunohistochemical receptor status, including estrogen receptor (ER), progesterone receptor (PR) and human epidermal growth factor receptor 2 (HER2). The tumors were classified into molecular subtypes via immunohistochemical surrogates.

### 2.4 Image analysis

The preoperative and postoperative MRIs were reviewed in consensus by two radiologists (JVH and KP) specialized in breast imaging with 6 and 12 years of experience, respectively. The radiologists were blinded for clinical data. The axillary and intramammary LNs ipsilateral to the operated breast were first identified on the postoperative MRI and then compared with the preoperative MRI. LNs were evaluated on non‐fat saturated T1‐weighted, fat saturated T1‐ and T2‐weighted, and contrast enhanced T1‐weighted sequences regarding the following: enlargement, cortical thickening, presence of fatty hilum, irregularity, heterogeneity, matting and axillary LN asymmetry. Additionally, measurements of the long axis and the cortical thickness on the largest axillary and intramammary LNs identified in each case were done on the slice where fatty hilum was best visualized. If there was a lack of a fatty hilum, the short axis was considered as the cortical thickness.

### 2.5 Statistical analysis

Statistical analyses were performed using SAS statistical software version 9.4 (SAS Institute, Cary, NC). Categorical variables were summarized using frequencies and percentages for categorical variables. Continuous variables were summarized using medians and ranges. Long axis and cortical thickness of LNs were presented as mean ± standard deviation measured in millimeters. Measurements were done on a “node‐by‐node” basis and bilateral nodes in the same patient were assumed to be non‐correlated. We assessed differences between groups using *t* tests and the log‐rank test. All tests were two sided and we considered *P* < 0.05 to be indicative of statistically significant differences.

## 3 RESULTS

There were 302 LNs detected in 51 axillae in 50 patients ipsilateral to the operated breast on both preoperative and postoperative MRIs with an average of 5.9 LNs per axilla (range, 2‐11). Patient and lesion characteristics are summarized in Table 1. The average time between surgery and the postoperative MRI was 224 days (range, 20‐356) and the average time between the preoperative and the postoperative MRI was 247 days (range, 39‐400).

**Table 1:** Patient and lesion characteristics

Characteristics of patients and lesions	N	%
Patient mean age 53 y (range, 32‐74)		
Total number of patients	50	100
Patients with breast implants	2	4
Treatment received prior to postoperative MRI		
Radiation therapy	45	90
Hormone therapy	46	92
Chemotherapy	15	30
Total number of breasts with primary tumors	51	100
Histology		
Invasive ductal carcinoma	47	92.2
Invasive lobular carcinoma	4	7.8
Tumor subtype		
Luminal A	46	90.2
Luminal B	1	2.0
HER2 enriched	0	0
Basal‐like	4	7.8

### 3.1 Axillary lymph nodes

Visual assessment of axillary LNs demonstrated that none of the patients presented with a new lack of a fatty hilum, irregularity, heterogeneity, matting or axillary LN asymmetry on postoperative MRI in comparison with the preoperative study. In 3/50 (6%) patients, 3/302 (1%) axillary LNs presented with enlargement and cortical thickening, with an average increase of 2.0 mm in the long axis and 1.9 mm in cortical thickness. All patients had 2‐year clinical follow‐up with conventional imaging and one patient had an additional MRI that ruled out recurrence.

**Figure 1:**
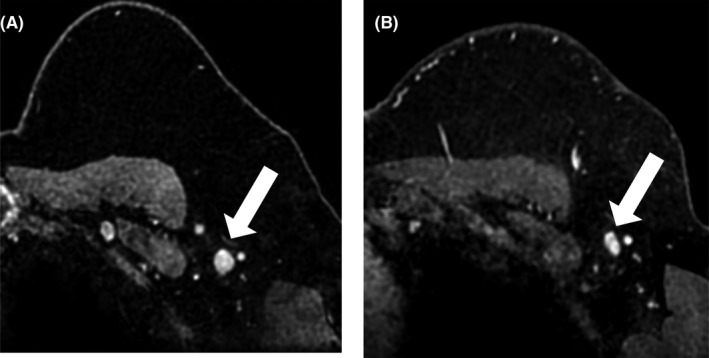
Change in morphology of LNs: T1‐weighted contrast enhanced images showing a 53‐y‐old woman with an axillary LN with lack of fatty hilum on preoperative MRI (A) with reduction in size on postoperative MRI (B, arrows)

**Figure 2:**
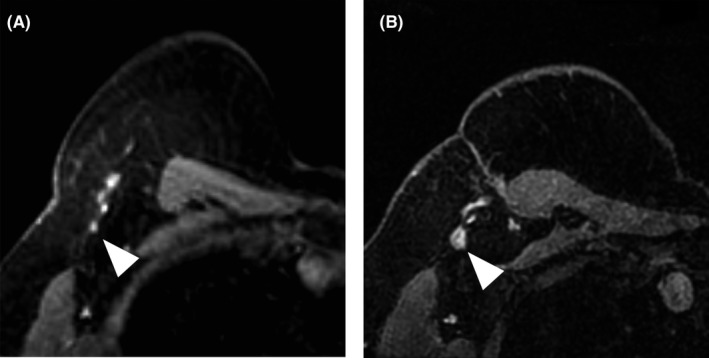
Change in morphology of LNs: T1‐weighted contrast enhanced images showing a 45‐y‐old woman with a small axillary LN on preoperative MRI (A) with enlargement on postoperative MRI (B, arrowheads)

The average long axis and cortical thickness of all axillary LNs on postoperative MRIs were 11.9 mm and 3.5 mm, respectively, while on preoperative MRIs were 12.5 mm and 3.7 mm, respectively. Whereas there was no statistically significant difference in cortical thickness on postoperative MRIs in comparison with preoperative MRIs (*P* = 0.106), there was a significant average reduction of 0.6 mm in the long axis observed in the postoperative MRIs (*P* = 0.029). Example cases of change in morphology are depicted in Figures 1 and 2.

### 3.2 Intramammary lymph nodes

**Figure 3:**
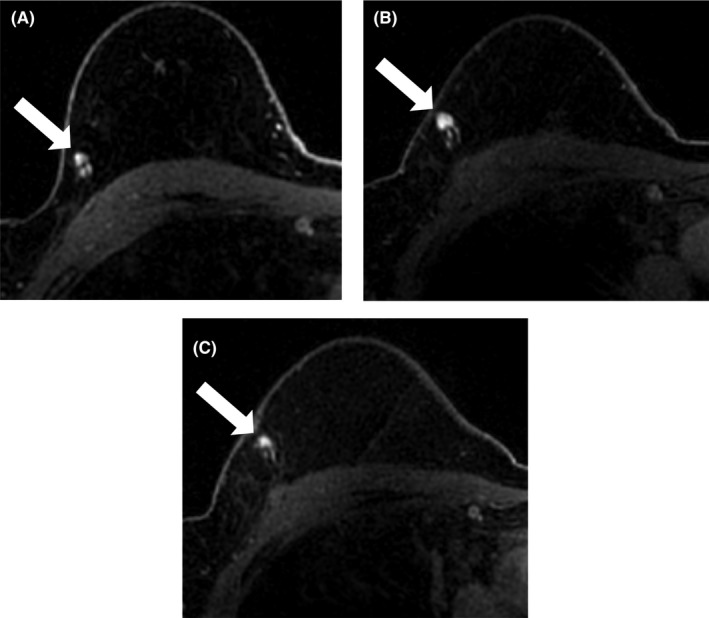
Change in morphology of LNs: 48‐y‐old woman with a normal appearing intramammary LN on preoperative T1‐weighted contrast enhanced image (A), presenting with enlargement on postoperative MRI (B). Follow‐up MRI (C) 2 y later showed that the LN returned to its preoperative dimensions

There were eight ipsilateral intramammary LNs detected in seven patients. Visual assessment demonstrated that none of the LNs presented a new absence of a fatty hilum, irregularity, heterogeneity, or matting on postoperative MRI compared with the preoperative study. In 2/7 (28.6%) patients, 2/8 (25%) LNs presented with enlargement and cortical thickening, with an average increase of 1.4 mm in the long axis and 1.7 mm in cortical thickness. All of these patients had 2‐year clinical and imaging follow‐up, including MRI, with no evidence of recurrence (Figure 3).

There was no statistically significant difference in the mean long axis and the cortical thickness of intramammary LNs on postoperative MRIs in comparison with preoperative MRIs (*P* = 0.302 and 0.809, respectively). The average long axis and cortical thickness of intramammary LNs on the postoperative study were 7 and 3.2 mm, while on the preoperative MRI they were 6.7 and 3.1 mm respectively.

Overall, enlargement and cortical thickening were seen in 5/50 (10%) patients. No LNs demonstrated occurrence of lack of a fatty hilum, irregularity, heterogeneity, matting, or axillary LN asymmetry. No evidence of recurrence was observed at 2‐year follow‐up.

## 4 DISCUSSION

No significant changes in the imaging features of axillary and intramammary LNs on postoperative MRIs were observed in the vast majority of patients after breast‐conserving surgery and adjuvant radiation therapy in breast cancer patients with negative axilla in their first year after surgery. A new lack of fatty hilum, cortical irregularity, heterogeneity, matting, or axillary LN asymmetry were not seen in any of the 310 LNs evaluated. Enlargement and cortical thickening of LNs were observed in 10% of patients and were not related to malignancy in our series as no signs of recurrence were observed at 2‐year follow‐up.

Several studies have investigated the features of LN on preoperative MRI that indicate metastatic infiltration.^12^, ^13^, ^14^, ^15^, ^16^, ^17^, ^18^, ^19^, ^20^, ^21^, ^22^, ^23^, ^24^ Although nodal size, cortical thickening, absent fatty hilum, irregularity, heterogeneity, matting, and asymmetry to the opposite axilla can be used to diagnose nodal metastasis, there is significant overlap between the imaging features of benign and malignant nodes. To this date, there is no consensus on which LN features should be used to characterize malignancy; thus, not rarely, the radiologist faces a diagnostic dilemma when evaluating LNs on MRI.

Cortical thickness is one of the most investigated features used to characterize nodal malignancy on MRI.^4^, ^16^, ^17^, ^20^, ^26^ A study conducted by Korteweg et al^16^ demonstrated that a 3 mm cut‐off point for cortical thickness had a sensitivity of 88% and a specificity of 32%, while another study by Luciani et al^17^ used a 4 mm cut‐off point and found a sensitivity of 78.6% and a specificity of 62.3%. Size and size/cortical thickness ratio were also investigated to differentiate benign from malignant nodes, but again results demonstrated significant overlap.^20^ Another feature frequently investigated is the presence of a fatty hilum on LNs.^18^, ^20^ Although the absence of fatty hilum is frequently seen in metastatic LNs, this can also be seen in up to one‐third of benign LNs.^16^ Baltzer et al^15^ demonstrated that cortical irregularity and axillary LN asymmetry had a very high specificity in diagnosing nodal metastasis. However, data indicate that these features are often absent in patients with metastatic nodal disease.^27^, ^28^

Whereas the majority of studies focused on the preoperative setting, there are scarce data on the imaging features of LNs on MRI in the postoperative period. Kim et al investigated the morphology of axillary LNs on ultrasound in the postoperative period.^25^ The authors reviewed 1796 studies from 874 asymptomatic patients after mastectomy and found that only 22 suspicious LNs were detected on surveillance ultrasound, six of which represented nodal metastasis on biopsy. In our study, we also showed that the incidence of abnormal LNs after surgery for breast cancer is low and that the majority of suspicious LNs detected in the postoperative period are benign.

In our study, no occurrence of lack of fatty hilum, irregularity, heterogeneity, matting, or axillary LN asymmetry was observed. Increase in long axis and cortical thickness of axillary and intramammary LNs occurred in 10% of cases, which most likely represented benign sequelae of surgery and adjuvant treatment, since no recurrence was detected at 2‐year clinical and imaging follow‐up. These results indicate that in patients with negative axilla and LN enlargement in the first year after surgery, short‐term imaging follow‐up may be an adequate alternative to LN biopsy.

Our retrospective study has some limitations. Only one patient had an MRI study within 3 months after surgery; thus, insights into the early postoperative period are limited. We only included patients with clinically negative axilla and breast‐conserving surgery; thus, our results should only be considered relevant for this specific population. In addition, our relatively small population can also be seen as one limitation; thus, prospective studies with a larger number of patients are needed to better understand the imaging aspects of LNs on MRI in the postoperative period. Lastly, even though the postoperative MRIs were performed during or after completion of adjuvant treatment, and patients with postoperative nodal enlargement showed no signs of recurrence on 2‐year follow‐up, there is a slight possibility that some LN enlargements could be attributed to metastatic disease in patients with false negative sentinel LN biopsy.

In conclusion, LN enlargement and cortical thickening may be observed in a few patients in the postoperative period. In patients with clinically negative axilla, these changes in morphology are often related to treatment and favor short‐term follow‐up as an alternative to LN biopsy.
